# Medical student education program in Alzheimer’s disease: *The PAIRS Program*

**DOI:** 10.1186/1472-6920-12-80

**Published:** 2012-08-21

**Authors:** Angela L Jefferson, Nicole G Cantwell, Laura K Byerly, Darby Morhardt

**Affiliations:** 1 Vanderbilt Memory & Alzheimer's Center, Department of Neurology, Vanderbilt University Medical Center, Nashville, TN, USA; 2 Alzheimer’s Disease Center & Department of Neurology, Boston University School of Medicine, Boston, MA, USA; 3 Oregon Health & Science University School of Medicine, Portland, OR, USA; 4 Cognitive Neurology and Alzheimer’s Disease Center, Northwestern University Feinberg School of Medicine, Chicago, IL, USA

**Keywords:** Experiential learning, Qualitative methods, Communication, Dementia, Alzheimer's disease, Medical education, Service learning

## Abstract

**Background:**

As life expectancy increases, dementia incidence will also increase, creating a greater need for physicians well-trained to provide integrated geriatric care. However, research suggests medical students have limited knowledge or interest in pursuing geriatric or dementia care. The purpose of this study is to evaluate the *PAIRS Program* and its effectiveness in enhancing medical education as a service-learning activity and replication model for the Buddy Program^TM^.

**Methods:**

Between 2007 and 2011, four consecutive classes of first year Boston University School of Medicine students (n = 45; 24 ± 3 years, 58% female, 53% White) participated in a year-long program in which they were paired with a patient with early-stage Alzheimer’s disease (AD). Assessments included pre- and post-program dementia knowledge tests and a post-program reflective essay.

**Results:**

Program completion was 100% (n = 45). A paired-sample *t*-test revealed a modest improvement in dementia knowledge post-program (p < 0.001). Using qualitative coding methods, 12 overarching themes emerged from the students’ reflective essays, such as observing care partner burden, reporting a human side to AD, reporting experiences from the program that will impact future clinical practice, and obtaining a greater understanding of AD.

**Conclusions:**

Quantitative and qualitative findings suggest that the *PAIRS Program* can enhance the acquisition of knowledge, skills, and positive attitudes regarding geriatric healthcare in future generations of physicians, a skill set that is becoming increasingly relevant in light of the rapidly aging population. Furthermore, results suggest that The Buddy Program^TM^ model can be successfully replicated.

## Background

Alzheimer’s disease (AD) affects an estimated 5.3 million older Americans [1]. While there are a number of pharmacological and non-pharmacological approaches that have been shown to effectively treat AD symptoms, there is no proven treatment to prevent, arrest, or reverse pathophysiological changes associated with the disease [2-5]. Given the complexity of AD diagnosis and management, it is not surprising that approximately one third of general practitioners find clinically managing dementia more frustrating than rewarding [6] and a similar number report the dementia diagnosis is the responsibility of a specialist [6]. However, older adults receive the majority of their healthcare from primary care physicians [7], who report pessimistic attitudes toward dementia care [8], including difficulty establishing a definitive diagnosis [9], discomfort discussing diagnosis and care options [6], and lack of community and social service referral information [9,10]. Unfortunately, a majority of medical students report similar barriers with limited knowledge about aging [11], mixed attitudes toward older adults [12,13], and limited interest in pursuing geriatrics or dementia care [13,14]. Early exposure may increase comfort with older patients [15,16].

The Boston University *Partnering in Alzheimer’s Instruction Research Study (PAIRS) Program* is one unique service-learning program aimed at enhancing geriatric healthcare issues in medical education. The program’s student-focused objectives include: (1) educating students about AD and related cognitive impairment; (2) familiarizing students with care and support-related issues patients and their families encounter; (3) improving students’ communication skills with elders and cognitively impaired individuals; and (4) introducing students to career opportunities in geriatrics and related fields.

The *PAIRS Program* is the first educational initiative to replicate the Northwestern University Buddy Program^TM^. The current study aims to quantitatively and qualitatively evaluate the *PAIRS Program* in fulfilling the four learning objectives outlined above. We hypothesized that dementia knowledge would quantitatively increase post-program and that qualitative analysis of end-of-the-year reflective essays would yield themes aligned with the *PAIRS Program* objectives.

## Methods

### Program design

Starting in 2007 and repeating annually, first year Boston University medical students were invited to apply to the *PAIRS Program*, which included a written application and interview process. In September, students were selected for participation based on enthusiasm, professionalism, and commitment level for the program requirements across the academic year. Selected students completed pre-program dementia knowledge tests upon program enrollment (i.e., prior to training). Students then participated in three hours of formal lectures on AD and dementia fundamentals and communication skills for interacting with aging and cognitively impaired adults. Students were paired with an early-stage AD “buddy” (taking into consideration shared interests and geographical proximity) and introduced to their “buddy” at a Match Day event in October. Between November and May, the “pairs” met monthly for a minimum of four hours and participated in social and cultural activities, such as dinner or visiting a museum. Students reported reactions via activity journal entries and attended monthly luncheons to receive supplemental education from guest lecturers (e.g., AD diagnostics, neuropathology) and share experiences with each other and program staff. Students completed post-program dementia knowledge tests following the final program meeting in May. Students received elective credit upon completion. The Institutional Review Board approved the use of program data for research and determined the research was exempt from written consent in accordance with federal regulation 45 CFR 46.101(1). While written consent was not obtained, all students provided assent to allow their de-identified program data to be used for research purposes.

### Measures

#### The Buddy Program^TM^ Dementia Knowledge Test

A 33-item measure assessed dementia knowledge pre- and post-program [17]. Scores range 0–33 with higher scores indicating increased knowledge.

#### The Boston University PAIRS Program Dementia Knowledge Test

In 2009, a 64-item measure was developed in an effort to more comprehensively assess dementia knowledge pre- and post-program. This measure was first implemented at the beginning of program year 3 and therefore completed by a subset of students enrolled in program years 3 and 4. Scores range 0–64 with higher scores indicating increased knowledge.

#### Reflective essays

Students write an end-of-the-year essay reflecting on their *PAIRS Program* experiences, including what they knew about AD before beginning the program, what they learned during the program, and how their participation will affect their medical career.

### Data analyses

Descriptives were generated for the student applicants and participants. Pre- and post-program knowledge scores were compared using paired-sample t-tests with a post-hoc item analysis. Analyses were conducted using SPSS 16.0 (Chicago, IL). Significance was set at p < 0.01 for primary analyses and p < 0.001 for post-hoc comparisons.

To qualitatively analyze the essays, Consensual Qualitative Research strategies were employed [18]. Two staff (LB, NC) independently reviewed the essays from program years 1 and 2 (n = 22), developed major themes (domains), and coded content according to domains. The program director (AJ) provided feedback on overlap and discrepancies. Next, the coders came to a consensus on a final list of domains and core ideas within each domain. Finally, essays from program years 3 (n = 11) and 4 (n = 12) were analyzed by the two coders to verify that existing domains were saturated (i.e., no additional information was found that contributed to the understanding of the existing domains) [19].

## Results

### Student characteristics

Between 2007 and 2011, 4 program years were completed with 79 student applicants and 45 enrollees out of 700 total medical students enrolled between 2007 and 2010. Comparison of admitted (n = 45) versus not admitted applicants (n = 34) revealed no between-group differences for age (t_(62)_ = −1.32, p = 0.19), sex (*x*^2^ = 0.47, p = 0.49), education (t_(77)_ = −0.47, p = 0.64), prior professional experience with an individual with AD (*x*^2^ = 0.03, p = 0.87), or personal/familial familiarity with AD (*x*^2^ = 0.96, p = 0.33). See Table 1.

**Table 1:** Sample characteristics

	**PAIRS program participants n = 45**	**Applicants not admitted n = 34**	**p-value**
Age, years	24 ± 3	23 ± 3^†^	0.19
Sex, % female	58	50	0.49
Race, % White	53	--	--
Ethnicity, % Hispanic/Latino	3	--	--
Education, years	16 ± 1	16 ± 1	0.64
Professional experience with AD patient prior to program, % yes	49	47	0.87
Personal/familial familiarity with AD prior to program, % yes	33	44	0.33
Monthly interactions, total	5 ± 1	--	--
Remained in contact with buddy post-program, % yes	62	--	--

Note. Data presented as mean ± SD or %; blank cells (−−) reflect that information was not collected or did not apply to applicants not admitted to the program; ^†^ = age information was available on a subset of applicants (n = 19).

### Dementia knowledge

Dementia knowledge test performance at program entry as assessed by the Buddy Program knowledge test was unrelated to students’ prior professional experience with AD (t_(43)_ = 0.47, p = 0.64) or personal familiarity with AD (t_(43)_ = 0.09, p = 0.93).

A post-program statistical improvement in knowledge was observed (t_(44)_ = −6.3, p < 0.001). Inspection of mean pre- and post-program performances suggests an improvement of 2.5 items, corresponding to 7.5% enhanced accuracy (see Table 2). Post-hoc examination of items with the greatest improvement (i.e., p-values <0.001) suggests two key areas of increased learning. Students increased their knowledge about autopsy as the only method for a definitive diagnosis for AD (i.e., 36% accuracy pre-program versus 73% accuracy post-program) and the misconception that dementia is untreatable (i.e., 64% accuracy pre-program versus 91% accuracy post-program).

**Table 2:** Dementia knowledge test performance

	**Pre-program**	**Post-program**	**p-value**
Buddy Program Knowledge Test^†^	26.5 ± 2.4	28.9 ± 1.9	<0.001
PAIRS Program Dementia Knowledge Test^‡^	40.4 ± 5.5	44.9 ± 4.3	<0.001

Note. Data presented as mean ± SD; ^†^ = analyses based on n = 45 participants; ^‡^ = analyses based on n = 23 participants.

A post-program improvement in knowledge was also observed for the subset of participants (n = 23) who completed the Boston University PAIRS Program Dementia Knowledge Test (t_(22)_ = −5.1, p < 0.001). Inspection of mean pre- and post-program performances suggests an improvement of approximately 4.5 items, corresponding to 7% enhanced accuracy (see Table 2). Post-hoc examination of items with the greatest improvement in accuracy (i.e., p-values <0.001) suggests several key areas of enhanced knowledge. Students increased their understanding of the symptoms of “sundowning” (i.e., 44% accuracy pre-program compared to 76% accuracy post-program) and their knowledge about risk factors for AD, such as educational attainment (i.e., 26% accuracy pre-program compared to 51% accuracy post-program). Similar to the Buddy Program Knowledge Test, students enhanced their knowledge about autopsy as the only definitive method for diagnosing AD (i.e., 39% accuracy pre-program compared to 74% accuracy post-program).

### Reflective essays

Essay themes are summarized in Table 3 and described below.

**Table 3:** Reflective essay themes

**Theme**	**Description**
Reasons for joining *PAIRS Program*	Reasons why students became interested in and decided to join the *PAIRS Program*
Previous scientific knowledge of AD	Students' knowledge of the pathophysiology and treatment modalities of AD prior to participation in the program
Previous personal experience with AD patient	Students’ previous experience interacting with an individual with AD prior to participation in the program
Initial impressions of buddy	Students' initial impressions of buddy and concerns for their initial interaction
Observation of AD symptoms	Interactions with their buddy allowed students to observe symptoms of AD in the home and in public settings
Greater understanding of AD	Through participation in the *PAIRS Program*, students gained a deeper, more well-rounded understanding of AD.
Care partner burden	Student’s perception of how the care partner deals with their loved one's diagnosis, the role the care partner plays in the buddy’s life, and the emotional and physical toll of providing such care
Human side of AD	The students now view AD as a total life-changing situation, not just a clinical diagnosis, and learned to address the person in addition to the symptoms of the disease
Buddy and care partner's hopeful outlook towards having AD	The buddy and care partner’s optimistic approaches to coping with AD and maintaining a fulfilling life
Educational value of monthly program meetings	Monthly program luncheons provided an educational forum and the opportunity to learn through the shared experiences of fellow students
Program impact on clinical practice	Students feel the program has changed the way they will practice as physicians, impacting their attitudes toward patients and their approach to providing care
Influence on medical specialization	*PAIRS Program* experiences have influenced students' decisions to specialize in AD related fields, such as neurology or geriatrics

#### Reasons for joining program

Students joined the program to enhance their understanding of AD and develop clinical skills. One student explained: “I [joined]…because of the unique learning experience that it offered students to become involved outside of the classroom.” Another student wrote:

“I wanted to have some positive experiences with Alzheimer’s patients especially since I am interested in Geriatrics. I knew that [AD] impacts many people in the geriatric population and will continue to have a growing impact and therefore it was important to me to learn how to effectively communicate with this population.”

#### Previous scientific knowledge of AD

Previous AD knowledge was scientific and academic in nature. One student explained: “I didn’t know much about [AD] except for what I learned in school - memory loss, tau protein, tangles…a scientist view.” Another student wrote, “Prior to joining…I had a vague idea of the pathophysiology, but I had no knowledge at all regarding the personal and social impact of [AD].”

#### Previous experience with AD patient

Some students had limited prior experience with AD. One student wrote: “I had never met anyone who had [AD] or who was a caregiver for someone with it.” Students with personal familiarity described their experience:

“I can remember visiting [my grandmother] at the nursing home…at first she was very sociable, but after a few years…minor issues, such as turning off the bathroom light, progressed to major issues, such as remembering where she was…finally the day came when she forgot who I was.”

#### Initial impressions of buddy

Students had preconceived notions about their buddies. One student wrote: “At first, I did not see any major deficits. This was not the picture of Alzheimer’s that had been painted for me in my Biochemistry courses.” One student admitted: “My expectations of [his] capabilities were low…according to my initial perception of what AD was, he blew me out of the water every time I met with him.” One student confided: “I was nervous…about hitting it off with my buddy and being able to spend so much time with him, but we connected really well and I greatly looked forward to our outings.”

#### Observation of AD symptoms

One student reported: “I hadn’t known that long-term memory stays largely intact. [He] could tell me all about his days in the U.S. Navy…in pretty astonishing detail, but he couldn’t tell me what he had for breakfast.” Another student recalled: “He spent a lot of time trying to get the camera to work. At times the camera was upside down, or he would press the on/off button instead of the snapshot button.” Another student explained: “[He makes] inappropriate [jokes] or misunderstood jokes with strangers because he has forgotten the context.”

#### Greater understanding of AD

Through formal and experiential learning, students gained a deeper understanding of AD. One student remarked: “Before I started…I did know about different aspects of Alzheimer’s but not in any context; the program helped tie all my knowledge together while providing much more data – both scientific and experience related.” One student concluded: “[This] has been an incredible experience for me to learn about how far [AD] permeates into a person’s life, far beyond the signs and symptoms that the physician or neuroscientist witnesses.”

#### Caregiver burden

Students reported observing the physical and emotional strain of the caregiver. One student reported: “I hadn’t realized how draining being a caregiver can become. We hear about the strain on caregivers all the time, but it is another thing to see it right in front of you.” A student wrote: “At one point [my buddy’s wife] told me that he was a different person. It’s hard to completely understand what that’s like, in a way she was losing him while he was still there.”

#### Human side of AD

Witnessing the everyday impact of AD on the patient’s life revealed a humanistic side of the disease. One student wrote: “My buddy allowed me to see Alzheimer’s through his eyes [and] to better understand his difficulties, frustrations and concerns.” Another student reported: “It’s one thing to study how proteins in the brain affect cognitive functioning, it’s quite another to see how families deal with a fading husband or father.” One student stated: “As a first year medical student, I am so glad I had the opportunity to witness firsthand the personal side of a disease. Classes this year focused on scientific study of disease, but my personal experiences seem equally important.” Another student wrote:

“Medical students don’t always get such personal accounts, yet such experiences enrich our understanding [so] that we remember for future patients to treat the person as well as the disease, a concept that…sometimes is lost.”

#### Buddy and caregiver's hopeful outlook

Students were surprised by their buddy and family’s optimistic approach for coping with AD. Students wrote: “[His] upbeat attitude despite his often depressing illness was refreshing and motivating” and “[My buddy] is one of the strongest people I have ever met and she continues to keep a positive attitude in the face of her terrible disease.” Another student wrote:

“The way [they] cope with this life-altering condition is very inspiring. Instead of being consumed by negative feelings, they actively took charge of the huge transition in both of their lives. They both play active roles in the Alzheimer’s Association. [My buddy] spoke…on TV about the disease. But he did not attain his current optimism without struggle.”

#### Monthly program meetings

Students reported benefits of the monthly meetings. One student wrote: “I’ve learned so much about AD not just from my buddy but also from my fellow [program] classmates…who have shared their experiences and their reactions in our monthly meetings.” Another student wrote: “Discussing and sharing stories about our buddies…has enlightened me on how each individual with AD experiences the symptoms in unique ways and how AD affects family members in different ways.”

#### Program impact on clinical practice

Students reported that participation changed their future practice and improved their attitudes towards elders. A student reflected: “I learned practical skills, such as communicating with elder patients…and how to treat patients with empathy and respect. I think that PAIRS is a valuable program and has made me a better overall healthcare provider and human being.” Another student noted: "The great doctor takes the time to assess the impact on the patient’s quality of life and also treats the emotional and social ‘symptoms’ of the problem.” One student explained: “This program has given me knowledge and experience that I believe will make me a better physician regardless of the field that I go into.”

#### Influence on specialization

For certain students, participation either solidified an existing interest or positively influenced their opinion of geriatrics or AD-related fields they may not have previously considered. Students wrote: “I entered the program with a slight interest in neurology, but I leave it with a significant one” and “The program…has certainly made me more comfortable working with older adults.” Another student wrote:

“I certainly have a stronger interest in [the older adult] patient population as a result of the program. I used to have this perception that it would be very difficult to work with older patients, especially those with dementia, but I realize now that although it takes a bit more effort, it is more than worthwhile.”

## Discussion

This study quantitatively and qualitatively evaluated the *PAIRS Program*’s impact on first year medical student education. Results indicate quantitative improvements in dementia knowledge that are unrelated to prior professional or personal exposure to AD, offering support of the program’s first objective of increasing student knowledge about AD and related cognitive impairment. Qualitative essay results further support this finding, as students reported directly observing cognitive and behavioral symptoms, such as difficulties with memory, verbal fluency, communication skills, and inhibition. The buddy interactions were augmented by group discussion and guest lecturers during monthly meetings with program leadership and staff. Because each student formed a unique relationship with a different patient, discussion of experiences exposed students to multiple perspectives and situations not encountered with their own buddy. Students reported that the guest lecturers covering neuropathology, diagnostic advances, and emerging therapeutic targets complimented their experiential learning. Collectively, these data imply that a combination of formal and experiential education facilitated acquisition of a deeper understanding of AD.

Qualitative findings further support the program’s second objective to enhance student awareness of care and support-related issues encountered by patients and their families. By establishing a personal relationship, students were exposed to the psychosocial challenges of coping with a chronic disease that extend beyond a prescribed set of symptoms. Students reported witnessing the psychosocial and physical impact of caregiving, reflecting recognition of the disease’s impact on the entire family system. Qualitative results suggest program participation increased students’ awareness of the human side of AD and their compassion for elders experiencing dementia.

Results from the reflective essays also support the third program objective, namely enhancing students’ communication skills and patterns when interacting with older individuals. Students reported feeling more comfortable with their communication skills and more confident that their newfound understanding of AD would translate into more compassionate and considerate care of elders. Students reported improved attitudes toward older and cognitively impaired individuals and discussed the value of maintaining respect and optimism when delivering a diagnosis or recommendations. Finally, students described increased awareness of elder-centric healthcare needs and expressed increased interest in or comfort working with elders.

The fourth program objective was to introduce students to career opportunities in geriatrics and related fields. Some students reported that their buddy experiences favorably influenced or even confirmed their decision to specialize in geriatrics or neurology. Other students developed an interest in contributing to AD research, either during medical training or later in their career. Such specialization outcomes will require follow-up of the student cohort, as only 18 of the 45 student participants to date have completed the residency matching process.

Collectively, our quantitative and qualitative findings support the notion that the Buddy Program^TM^ model can be successfully replicated and that this program model enhances first year medical student educational experiences. The program structure compliments a recent progression of medical curriculum toward experiential learning initiatives. Contemporary medical school curricula are dissolving traditional divisions of pre-clinical and clinical training with a trend toward early exposure to practical experience and patient populations [20]. In fact, the Liaison Committee on Medical Education recently changed accreditation policies to incorporate service-learning, defined as a structured learning experience that combines community service with preparation and reflection. Medical schools must now provide sufficient opportunities for medical students to participate in service-learning. Our program model not only meets such service-learning criteria but can be replicated by other institutions to enhance their service-learning opportunities.

Innovative educational programs that enhance the acquisition of knowledge, skills, and positive attitudes regarding geriatric healthcare are more important than ever. As life expectancy increases, the demand for physicians qualified to handle complex geriatric healthcare will exceed physicians available [21]. Knowledge and skills in geriatric care, regardless of specialty, is essential to providing quality, cost-effective healthcare to elders with chronic conditions, including dementia. Therefore, core medical school training would benefit from geriatric-based programs and sensitization to geriatric issues to ensure competency among future physicians caring for older adults. As an example, senior mentor programs introduce students to healthy community-based elders to help students view aging as a multidimensional process [22]. Such programs have enhanced students’ attitudes towards elders [23,24], improved students’ communication skills [23], and taught students the value of interdisciplinary care [24]. Our findings suggest that the *PAIRS Program* and The Buddy Program^TM^ expand the fundamentals of existing mentor programs with a unique focus on needs of older adults with cognitive impairment. Therefore, in addition to fulfilling a service-learning need, the *PAIRS Program* responds to the need for medical school curriculum to improve attitudes toward elders.

The present study has several strengths. Our program is the first formal replication of The Buddy Program^TM^, and our quantitative and qualitative outcomes support the reproducibility of the model for fulfilling the student training objectives put forth. Second, while experiential educational programs between students and elders currently exist, these programs emphasize healthy older adults, often screened to exclude individuals with chronic disease or cognitive impairment. The Buddy Program^TM^ and *PAIRS Program* model is among the first to exclusively expose medical students to cognitively impaired elders and represents a unique educational initiative responding to the increased prevalence of dementia and efforts to provide medical students with service-learning opportunities. Finally, the combination of qualitative and quantitative methodology provides a comprehensive examination of program data and extends prior outcomes from The Buddy Program^TM^[17].

In light of our small sample size (n = 45) and the purposeful selection of our students, generalizability of our findings to a larger medical student population may be limited. Because only students interested in the program goals elected to apply and the students selected were among the more enthusiastic and committed students who interviewed, it is possible that our participants’ engagement in the program does not reflect that of the average first year medical student. It is noteworthy that comparison of our participants with applicants not selected for participation suggests comparable pre-existing exposure to and personal/familial familiarity with AD. However, it is still plausible that all applicants, whether selected or not selected for participation, are fundamentally different from their classmates who did not apply for the program. Another noteworthy limitation is that while there was a modest improvement on the pre-and post-tests of Alzheimer’s disease and dementia knowledge, the source of improvement is unknown. That is, it is unknown if enhanced knowledge over the academic year was due to the monthly student luncheons with supplemental education, monthly buddy interactions, or some combination of these curricular elements. Themes from the student’s reflective essays support the notion that a combination of the monthly luncheons and the buddy interactions enhanced student knowledge; however, future evaluation methods should attempt to better understand the most valuable element of the program in enhancing student knowledge.

Future directions include continued assessment of the *PAIRS Program* and expansion of existing evaluation methods. We will continue to track the residency selections for program alumnae with each class of graduating students. In program year 5, we introduced quantitative assessment of changes in students’ attitudes, stigma, and empathy via survey tools pre- and post-program participation. These measures are designed to more comprehensively monitor the development of key elements of humanism in medicine. Research is also needed to evaluate prospective replication of the program model among other student cohorts, such as nurses, social workers, and undergraduate students. Finally, evaluation methods could be enhanced by addition of buddy and care partner feedback.

## Conclusion

This study evaluated the *PAIRS Program* and its effectiveness in enhancing medical education as a service-learning. Quantitative and qualitative analyses of data from the first four program years show post-program improvements aligned with the program objectives. Performance on knowledge tests and themes extracted from students’ reflective essays demonstrate an increase in dementia knowledge, appreciation for the psychosocial and support-related challenges faced by patients and families, enhanced communication skills, and exposure to dementia-related fields of medicine and research following completion of the program. Our findings suggest the *PAIRS Program* can successfully fulfill the need for service learning opportunities while improving positive attitudes regarding the healthcare of older or cognitively impaired adults. Increased interest in geriatric healthcare is more important than ever in light of the growing needs of our rapidly aging population. Moreover, our study supports the ability to successfully replicate The Buddy Program^TM^ model and encourages additional institutions to implement similar programs to enrich students’ medical education.

## Competing interests

The authors declare they have no competing interests.

## Authors' contributions

AJ and DM designed the study. NC and LB conducted qualitative analyses, and AJ conducted statistical analyses. AJ, NC, LB assisted with interpretation of results. AJ, NC, LB, and DM assisted with critical revision of the manuscript. All authors read and approved the final manuscript.

## Pre-publication history

The pre-publication history for this paper can be accessed here:

http://www.biomedcentral.com/1472-6920/12/80/prepub

## References

[B1] Association A's Alzheimer's disease facts and figures Alzheimers Dement 2010 2010 6 158 194 10.1016/j.jalz.2010.01.009 20298981

[B2] Farlow MR Miller ML Pejovic V Treatment options in Alzheimer's disease: maximizing benefit, managing expectations Dement Geriatr Cogn Disord 2008 25 408 422 10.1159/000122962 18391487

[B3] Hogan DB Progress update: Pharmacological treatment of Alzheimer's disease Neuropsychiatr Dis Treat 2007 3 569 578 19300586 PMC2656293

[B4] Lavretsky H Nguyen LH Innovations: geriatric psychiatry: diagnosis and treatment of neuropsychiatric symptoms in Alzheimer's disease Psychiatr Serv 2006 57 617 619 10.1176/appi.ps.57.5.617 16675752

[B5] Leifer BP Alzheimer's disease: Seeing the signs early J Am Acad Nurse Pract 2009 21 588 595 10.1111/j.1745-7599.2009.00436.x 19900220

[B6] Turner S Iliffe S Downs M Wilcock J Bryans M Levin E Keady J O'Carroll R General practitioners' knowledge, confidence and attitudes in the diagnosis and management of dementia Age Ageing 2004 33 461 467 10.1093/ageing/afh140 15271637

[B7] Gazewood JD Vanderhoff B Ackermann R Cefalu C Geriatrics in family practice residency education: an unmet challenge Fam Med 2003 35 30 34 12564861

[B8] Harris DP Chodosh J Vassar SD Vickrey BG Shapiro MF Primary care providers' views of challenges and rewards of dementia care relative to other conditions J Am Geriatr Soc 2009 57 2209 2216 10.1111/j.1532-5415.2009.02572.x 19943831 PMC3832192

[B9] Cody M Beck C Shue VM Pope S Reported practices of primary care physicians in the diagnosis and management of dementia Aging Ment Health 2002 6 72 76 10.1080/13607860120101158 11827625

[B10] Hinton L Franz CE Reddy G Flores Y Kravitz RL Barker JC Practice constraints, behavioral problems, and dementia care: primary care physicians' perspectives J Gen Intern Med 2007 22 1487 1492 10.1007/s11606-007-0317-y 17823840 PMC2219799

[B11] Fitzgerald JT Wray LA Halter JB Williams BC Supiano MA Relating medical students' knowledge, attitudes, and experience to an interest in geriatric medicine Gerontologist 2003 43 849 855 10.1093/geront/43.6.849 14704384

[B12] Hughes NJ Soiza RL Chua M Hoyle GE MacDonald A Primrose WR Seymour DG Medical student attitudes toward older people and willingness to consider a career in geriatric medicine J Am Geriatr Soc 2008 56 334 338 10.1111/j.1532-5415.2007.01552.x 18179490

[B13] Voogt SJ Mickus M Santiago O Herman SE Attitudes, experiences, and interest in geriatrics of first-year allopathic and osteopathic medical students J Am Geriatr Soc 2008 56 339 344 10.1111/j.1532-5415.2007.01541.x 18086123

[B14] Chua MP Tan CH Merchant R Soiza RL Attitudes of first-year medical students in Singapore towards older people and willingness to consider a career in geriatric medicine Ann Acad Med Singapore 2008 37 947 951 19082202

[B15] Alford CL Miles T Palmer R Espino D An introduction to geriatrics for first-year medical students J Am Geriatr Soc 2001 49 782 787 10.1046/j.1532-5415.2001.49156.x 11454118

[B16] Cankurtaran M Halil M Ulger Z Dagli N Yavuz BB Karaca B Ariogul S Influence of medical education on students' attitudes towards the elderly J Natl Med Assoc 2006 98 1518 1522 17019923 PMC2569732

[B17] Morhardt D Educating medical students on Alzheimer's disease and related disorders: An overview of the Northwestern University Buddy Program Dementia 2006 5 448 456 10.1177/147130120600500311

[B18] Hill C Knox S Thompson B Williams E Hess S Ladany N Consensual Qualitative Research: An update J Couns Psychol 2005 52 196 205

[B19] Creswell J Qualitative inquiry and research design: Choosing among five approaches 2007 2 Thousand Oaks: Sage Publications

[B20] Stewart T Alford C Introduction: Older adults in medical education–senior mentor programs in U.S. medical schools Gerontology & Geriatrics Education 2006 27 3 10 10.1300/J021v27n02_02 17023379

[B21] Besdine R Boult C Brangman S Coleman EA Fried LP Gerety M Johnson JC Katz PR Potter JF Reuben DB Caring for older Americans: the future of geriatric medicine J Am Geriatr Soc 2005 53 S245 S256 15963180 10.1111/j.1532-5415.2005.53350.x

[B22] Anderson MB A thematic summary of the geriatrics curricula at 40 U S Medical schools Acad Med 2004 79 S2 13 26 10.1097/00001888-200407001-00044 15240246

[B23] Bates T Cohan M Bragg DS Bedinghaus J The Medical College of Wisconsin Senior Mentor Program: experience of a lifetime Gerontol Geriatr Educ 2006 27 93 103 10.1300/J021v27n02_10 17023387

[B24] Roberts E Richeson N Thornhill JT Corwin SJ Eleazer GP The Senior Mentor Program at the University of South Carolina School of Medicine Gerontol Geriatr Educ 2006 27 11 23 10.1300/J021v27n02_03 17023380

